# Rapid and effective preparation of clonal bone marrow-derived mesenchymal stem/stromal cell sheets to reduce renal fibrosis

**DOI:** 10.1038/s41598-023-31437-7

**Published:** 2023-03-17

**Authors:** Sumako Kameishi, Celia M. Dunn, Masatoshi Oka, Kyungsook Kim, Yun-Kyoung Cho, Sun U. Song, David W. Grainger, Teruo Okano

**Affiliations:** 1 Cell Sheet Tissue Engineering Center (CSTEC), University of Utah, Salt Lake City, Utah USA; 2 Department of Molecular Pharmaceutics, Health Sciences, University of Utah, 30 South 2000 East, Salt Lake City, Utah 84112 USA; 3 Department of Biomedical Engineering, University of Utah, Salt Lake City, Utah USA; 4 Department of Nephrology, Tokyo Women’s Medical University, Tokyo, Japan; 5 SCM Lifescience Co., Ltd., Incheon, Republic of Korea; 6 Institute for Advanced Biomedical Sciences, Tokyo Women’s Medical University, Tokyo, Japan

**Keywords:** Mesenchymal stem cells, Stem-cell biotechnology, Tissue engineering

## Abstract

Allogeneic “off-the-shelf” mesenchymal stem/stromal cell (MSC) therapy requires scalable, quality-controlled cell manufacturing and distribution systems to provide clinical-grade products using cryogenic cell banking. However, previous studies report impaired cell function associated with administering freeze-thawed MSCs as single cell suspensions, potentially compromising reliable therapeutic efficacy. Using long-term culture-adapted clinical-grade clonal human bone marrow MSCs (cBMSCs) in this study, we engineered cBMSC sheets in 24 h to provide rapid preparation. We then sought to determine the influence of cBMSC freeze-thawing on both in vitro production of pro-regenerative factors and in vivo ability to reduce renal fibrosis in a rat model compared to freshly harvested cBMSCs. Sheets from freeze-thawed cBMSCs sheets exhibited comparable in vitro protein production and gene expression of pro-regenerative factors [e.g., hepatocyte growth factor (HGF), vascular endothelial growth factor (VEGF), and interleukin 10 (IL-10)] to freshly harvested cBMSC sheets. Additionally, freeze-thawed cBMSC sheets successfully suppressed renal fibrosis in vivo in an established rat ischemia–reperfusion injury model. Despite previous studies reporting that freeze-thawed MSCs exhibit impaired cell functions compared to fresh MSC single cell suspensions, cell sheets engineered from freeze-thawed cBMSCs do not exhibit impaired cell functions, supporting critical steps toward future clinical translation of cBMSC-based kidney disease treatment.

## Introduction

Mesenchymal stem/stromal cells (MSCs) have been applied in hundreds of clinical trials to date based on their therapeutic secretome and paracrine potency. Many different cytokines and growth factors are implicated in MSC-based cell therapies, including immunomodulatory factors (e.g., interleukin-10: IL-10, prostaglandin E2: PGE-2)^1–3^, anti-fibrotic factors (e.g., hepatocyte growth factor: HGF, bone morphogenetic protein 7: BMP-7)^4–6^, and angiogenic factors (e.g., vascular endothelial growth factor: VEGF, basic fibroblast growth factor: bFGF)^7–9^. Specifically, MSC’s reportedly high immunomodulatory capacity has motivated several ongoing clinical studies for immune-related diseases, such as graft-versus-host disease (GvHD), Crohn's disease, and severe acute pancreatitis^10,11^, all of which lack effective conventional pharmaceutical treatment alternatives. Current MSC administration strategies utilize conventional injection-based delivery of MSC single-cell suspensions, considered advantageous for treating systemic diseases. However, for localized diseases, it is essential to employ local cell transplantation methods to enhance cell engraftment and survival rates in the targeted site, thus increasing the potential for sustained cell-based delivery of therapeutic factors^12,13^.

Cell sheet technology uses commercial thermo-responsive cell culture dishes (TRCDs) grafted with the temperature-responsive polymer, poly(*N*-isopropylacrylamide), allowing scalable harvest of cultured cells as a single, confluent sheet^14^ via non-enzymatic temperature-mediated detachment. By avoiding the use of enzymatic-mediated culture, cell sheets retain innate instructive ECM and cell–cell interactions^15,16^ that facilitate direct cell sheet transplantation without use of sutures. Our group has recently reported characterization of cell sheets engineered from human clinical-grade MSCs and demonstrated that MSC sheet transplantation in vivo prolongs cell retention at target tissue sites compared to single-cell injections^17,18^. Furthermore, our group has recently reported that MSC sheet formation enhances cytokine production compared to single-cell conditions in vitro^19–21^. Preclinical applications of directly transplanted MSC sheets demonstrate pro-regenerative therapeutic efficacy across several disease models and various tissues^22^, such as the heart^23^, periodontal ligament^24,25^, bone^26^, skin^27^, and kidney^17^. Interestingly, previous studies suggest that some transplanted GFP-labeled MSCs may transdifferentiate into endothelial cells, pericytes, and other cell types to support neovascularization to regenerate damage tissues^22,23,27,28^.

Despite the ongoing promise of MSC therapies, including MSC sheet therapy, major obstacles preclude clinical translation of MSC therapy, including notable scientific, practical, and economic challenges. Allogeneic “off-the-shelf” MSC strategies defined under scaled quality-controlled cell production methods now address several current issues, with anticipated improvements in cost and potency per cell dose^29,30^. Human MSC source standardization, mass manufacturing under quality control, product distribution, and clinical dosing regimens must be addressed^29,30^. In this study, we employed long-term culture-adapted human clonal bone marrow stem (stromal) cells (cBMSCs): MSC primary lines derived from a single human MSC and that exhibit stable cell proliferative capability beyond passage 10 with high regenerative capacity and low immunogenicity^31–33^. Human cBMSCs are clinically available through good manufacturing practice (GMP) production based on safety tests, including in vivo toxicity, biodistribution analysis, tumorigenicity tests, and karyotyping^34^. Therefore, cBMSCs used in this study represent a new opportunity to produce a potent, sustainably cell-banked allogeneic product with increased homogeneity for future clinical use.

Reliable, cost-effective clinical delivery of allogeneic MSC products to patients requires improvements in cell storage, cell manufacture, and transplantation systems. Currently, freshly isolated MSCs, called "non-cryopreserved MSCs" in this study, are employed in preclinical animal studies, while banked MSC products ("freeze-thawed MSCs") are prepared and directly transfused in human clinical trials to reduce procedural complexity^10^. Discrepancies between MSC protocols for preclinical animal studies and human clinical settings contribute to inconsistent clinical outcomes^10,35,36^; therefore, quality control of freeze-thawed human MSCs is necessary to validate off-the-shelf MSC products.

Previous studies reported that tri-lineage differentiation potency and MSC surface marker expression are well-preserved after cryopreservation^37^. However, freeze–thaw cycles often compromise MSC viability^30,36,38^. In addition, impaired immunomodulatory and blood regulatory properties are commonly reported in freeze-thawed MSCs^35,38,39^. Given changes to MSC function caused by cryo-handling and freeze–thaw cycles^29,30^, distinct challenges associated with freeze-thawed MSCs must be addressed before their direct utilization in human clinical products.

**Figure 1:**
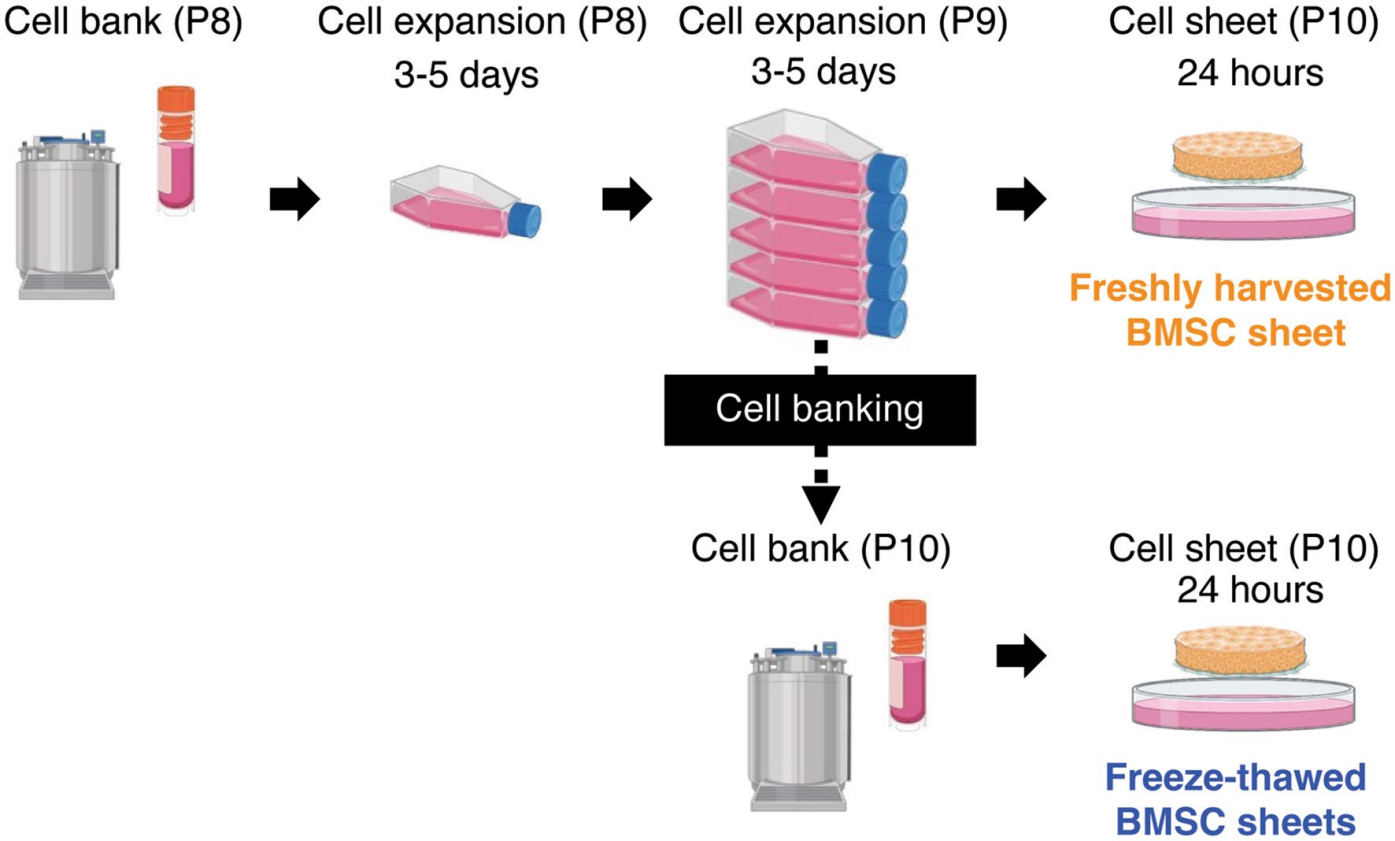
Preparation of freshly harvested and freeze-thawed clonal BMSC sheets. Clonal BMSC sheet preparation strategy using freshly harvested and freeze-thawed cells revived from each working cell bank. Cells were seeded onto thermo-responsive cell culture dishes (TRCDs) and cultured for 24 h to harvest as cell sheets.

Current MSC sheet production including cell expansion and cell sheet fabrication typically requires 2–3 weeks^19–21^ before application. Therefore, to reduce production time and associated costs, MSC sheet clinical translation would benefit from improved processes that utilize freeze-thawed MSCs from large-scale cell banks to yield rapid cell sheet fabrication while maintaining therapeutic effects. In this study, cell sheets were developed using GMP-grade cBMSCs^31,40^ certified by MSC criteria and currently investigated to treat GvHD in phase II clinical trials in Korea^41^. Cell sheets made from cBMSCs revived from cryo-banking, passaged twice and used for cell sheets (i.e., "freshly harvested cBMSCs") were compared to cell sheets from cBMSCs immediately revived from a working cell bank ("freeze-thawed cBMSCs") (Fig. 1). We compared cBMSC sheets from these two cell sources at identical passage numbers for pro-regenerative cytokine production in vitro and therapeutic suppression of renal fibrosis in an in vivo rat ischemia–reperfusion injury (IRI) model^17,42^, a potential clinical application. This study seeks to fill the gap between MSC preclinical model work and human clinical performance as an allogeneic cell therapy using freeze-thawed GMP-produced cBMSCs, ultimately required to improve MSC therapeutic and clinical translational impact.

## Results

### Evaluation of freshly harvested and freeze-thawed cBMSCs for cell viability and growth in vitro

**Figure 2:**
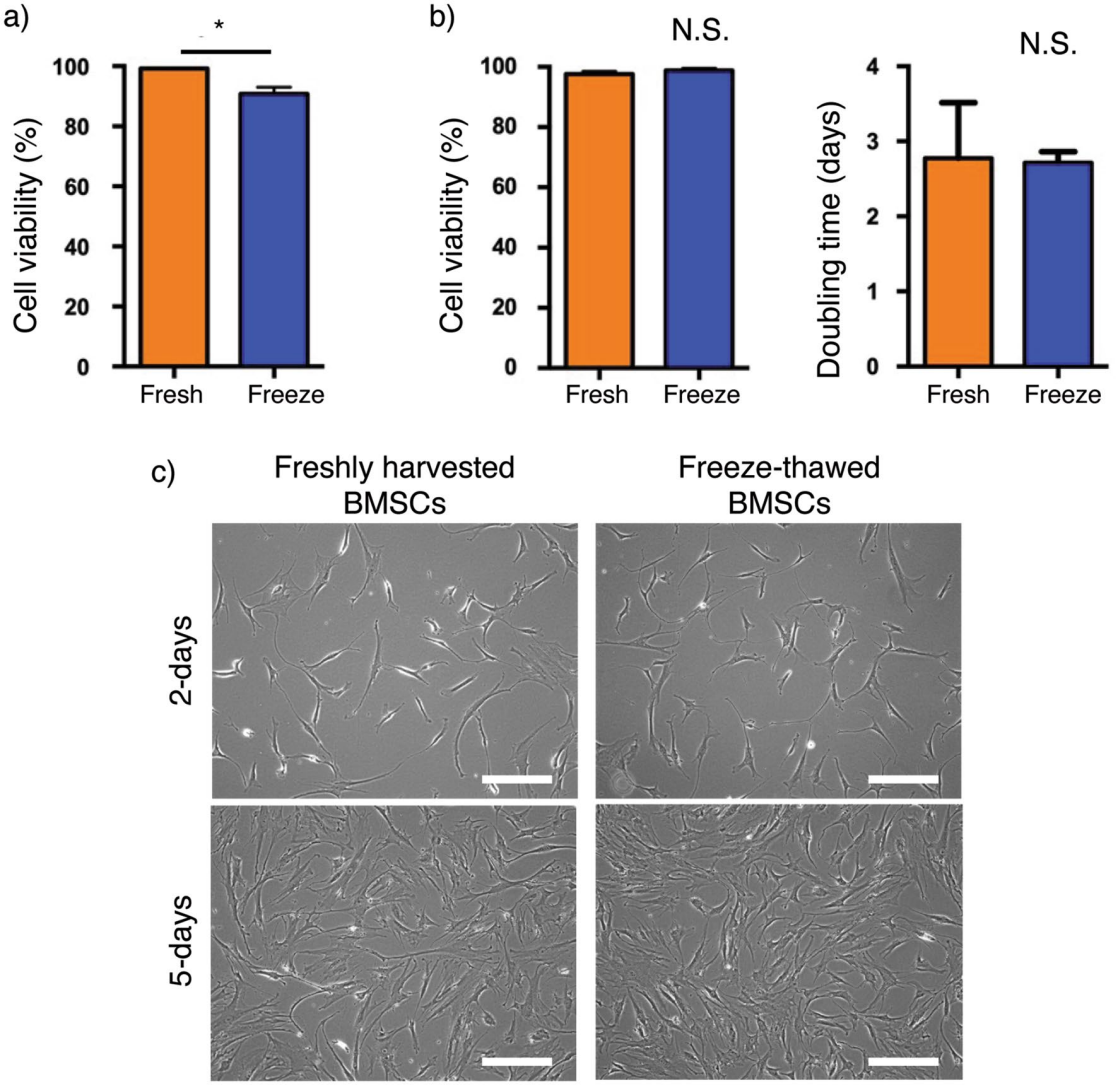
Cell viability and proliferation of freshly harvested and freeze-thawed human clonal BMSCs. (**a**) Cell viability of freshly harvested (Fresh) clonal BMSCs following culture and freeze-thawed (Freeze) clonal BMSCs following thawing from cell bank at passage 10, day 0. Data shown are mean ± SD (n = 4). Statistical significance: Student’s *t*-test, *P < 0.05. (**b**) Cell viability and doubling time of freshly harvested (Fresh) and freeze-thawed (Freeze) clonal BMSCs at passage 10, day 5. Data shown are mean ± SD (n = 4). Statistical significance: Student’s *t*-test, not significant (N.S.). (**c**) Phase-contrast images of freshly harvested and freeze-thawed clonal BMSCs at 2-day and 5-day culture. Scale bars represent 200 μm.

Viability of freeze-thawed cBMSCs was determined immediately following thawing from cryogenic banking and compared to freshly harvested cBMSCs revived from the same initial working cell bank but passaged twice (see Fig. 1). Freeze-thawed cBMSCs exhibited 90.8% cell viability, significantly lower than freshly harvested cBMSCs (99.1%, Fig. 2a). After 5 days of culture, both freshly harvested and freeze-thawed cBMSCs exhibited similarly high cell viability and growth rates (Fig. 2b) and became ~ 70% confluent after identical seeding conditions (Fig. 2c), indicating that freeze-thawed cBMSCs rapidly recover from cryopreservation. Cell size and morphology were comparable between the freeze-thawed and freshly harvested cBMSCs at both 2- and 5-day cultivation (Fig. 2c). These findings suggest that freeze-thawed cBMSCs adhere and proliferate equivalently to previously culture-rescued cBMSCs despite exhibiting reduced cell viability immediately following freeze-thawing.

### Cell adherence and spreading ability in freshly harvested and freeze-thawed cBMSC cultures in vitro

**Figure 3:**
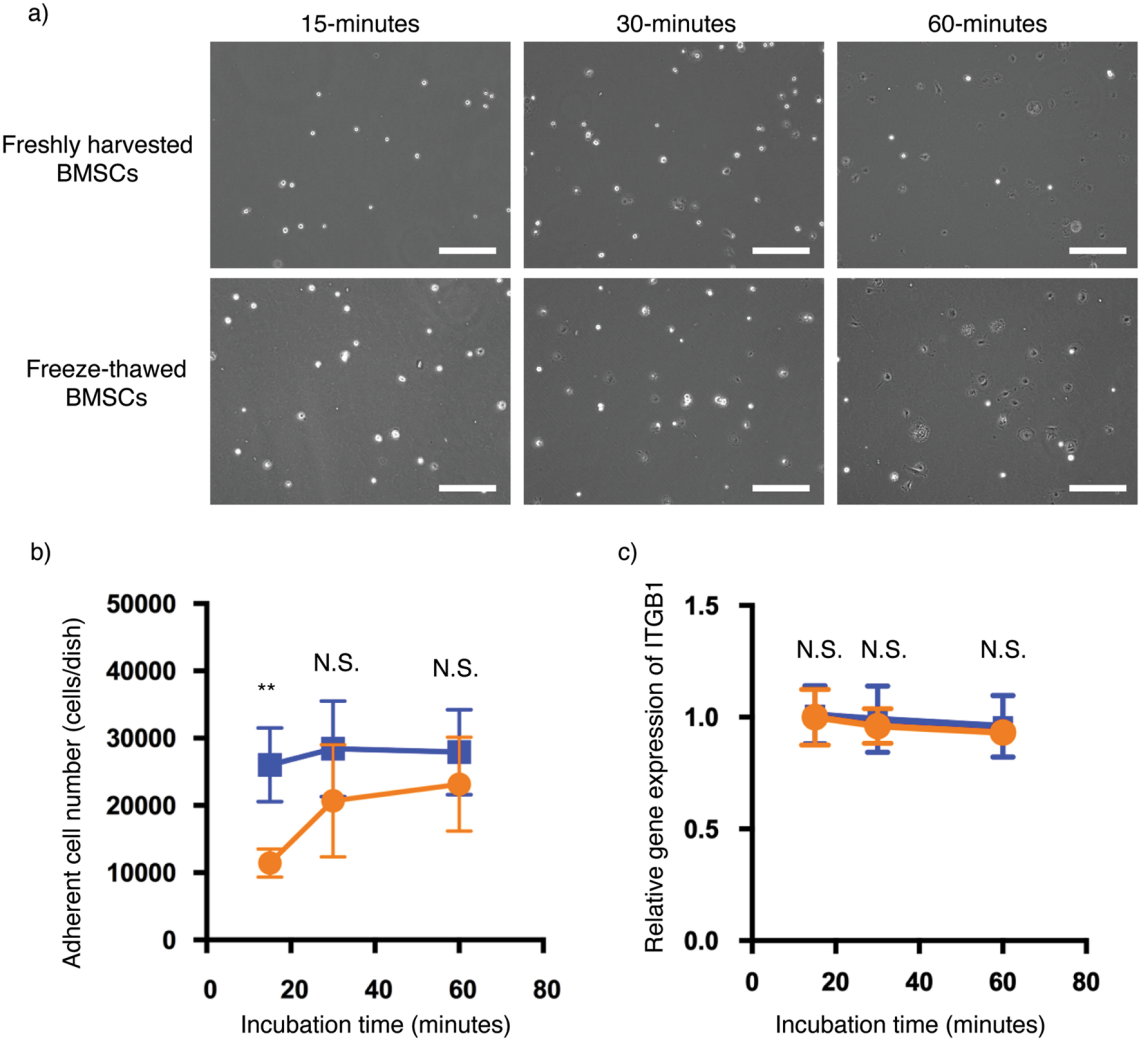
Cell spreading ability in freshly harvested and freeze-thawed human clonal BMSCs. (**a**) Phase contrast images of freshly harvested and freeze-thawed clonal BMSCs at 15-, 30-, and 60-min incubation after seeding in culture. Scale bars represent 200 μm. (**b**) Adherent cell numbers of freshly harvested (orange) and freeze-thawed (blue) clonal BMSCs are shown. Data shown are mean ± SD (n = 4). Statistical significance: Student’s *t*-test, not significant (N.S.), **P < 0.01. (**c**) Gene expression of *ITGB1* in freshly harvested (orange) and freeze-thawed (blue) clonal BMSCs were shown. Data shown are mean ± SD (n = 4). Statistical significance: Student’s *t*-test, not significant (N.S.).

Rapid cell sheet fabrication depends on rapid cell adhesion and spreading to form a confluent monolayer; therefore, cell adhesion and spreading properties of freeze-thawed cBMSCs are critically important to their ability to be used for cell sheet fabrication. Initial cell adhesion and spreading was observed after seeding freshly harvested and freeze-thawed cBMSCs onto cell cultureware and incubating for 15, 30, and 60 min. After incubation, cell culture dishes were washed with PBS to remove non-adherent cells (i.e., floating cells), and the remaining adherent cells were then observed. Interestingly, freeze-thawed cBMSCs exhibited increased cell spreading versus freshly harvested cBMSCs after both 15- and 30-min incubations, as shown in Fig. 3a. Additionally,  the number of adherent cells was approximately double in the freeze-thawed cBMSCs experimental group compared to freshly harvested cBMSCs after 15-min (Fig. 3b). Live cell time-lapse imaging further confirmed these trends, indicating that over time the freeze-thawed cBMSCs possessed higher intrinsic cell adhesion and spreading capabilities (Supplemental Figure S2 and Videos). To investigate differences during initial cell spreading processes, gene expression integrin β1, *ITGB1*, the primary cell adhesion receptor, was evaluated using qRT-PCR. No significant differences in integrin β1 gene expression were observed between freshly harvested and freeze-thawed cBMSCs after 10- and 60-min incubations, as shown in Fig. 3c. These findings indicate that freeze-thawed cBMSCs possess higher initial cell spreading and adhesion capability than freshly harvested cBMSCs, regardless of *ITGB1* expression.

### Adherent cell shape correlates to actin organization and focal adhesion formation during maturation of cell adhesion in culture

**Figure 4:**
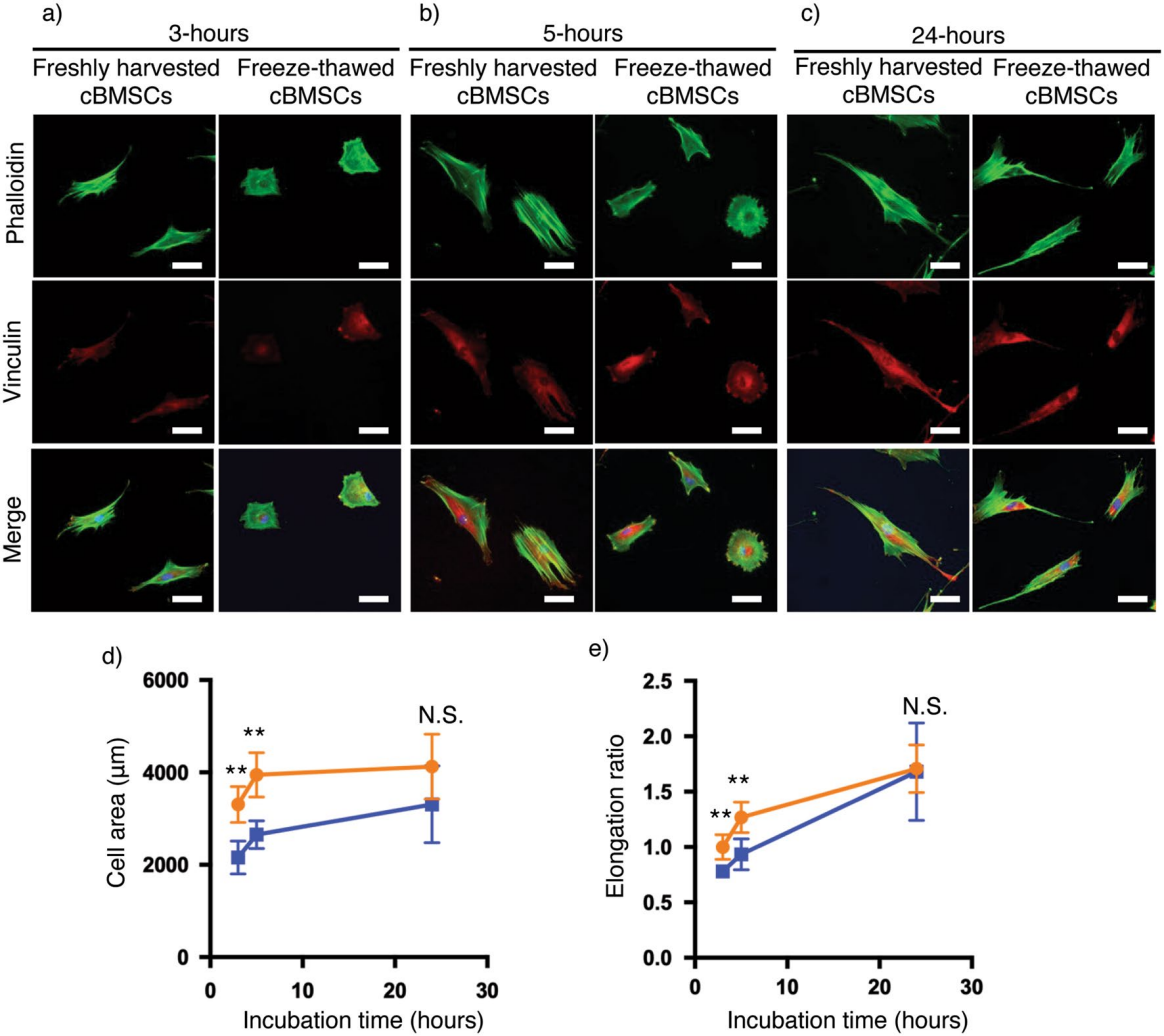
Actin organization and focal adhesion formation in freshly harvested and freeze-thawed human clonal BMSCs. (**a–c**) Fluorescent images of freshly harvested and freeze-thawed clonal BMSCs at 3-, 5-, 24-h incubation after seeding respectively. Scale bars represent 50 μm. (**d**) Cell area measurements of freshly harvested (orange) and freeze-thawed (blue) clonal BMSCs. Data shown are mean ± SD (n = 4). Statistical significance: Student’s *t*-test, not significant (N.S.) *P < 0.05. (**e**) Cell elongation measurements of freshly harvested (orange) and freeze-thawed (blue) clonal BMSCs. Data shown are mean ± SD (n = 4). Statistical significance: Student’s *t*-test, not significant (N.S.) *P < 0.05.

Immunohistochemistry (IHC) staining of vinculin, a focal adhesion marker, was performed using F-actin phalloidin (Fig. 4a–c), and cell morphology of adherent cells at each culture time point was quantified using Image J, as shown in Fig. 4d,e^43,44^. Freshly harvested cBMSCs exhibited larger average adherent cell size and more elongated cell shape compared to freeze-thawed cBMSCs at both 3- and 5-h time points (Fig. 4a,b). These differences faded by the 24-h time point (Fig. 4c). Additionally, increased vinculin localization uniformity was observed at the cell edges, co-localizing with F-actin fibers, in the freshly harvested cBMSCs compared to freeze-thawed cBMSCs at the 3-h time point (Fig. 4a, overlaid fluorescent images). These findings indicate that freshly harvested adherent cBMSCs develop more mature focal adhesions and elongated cell morphologies consistent with stromal phenotypes compared to freeze-thawed cBMSCs after seeding and culture.

### Cell sheet fabrication and cytokine production in culture

**Figure 5:**
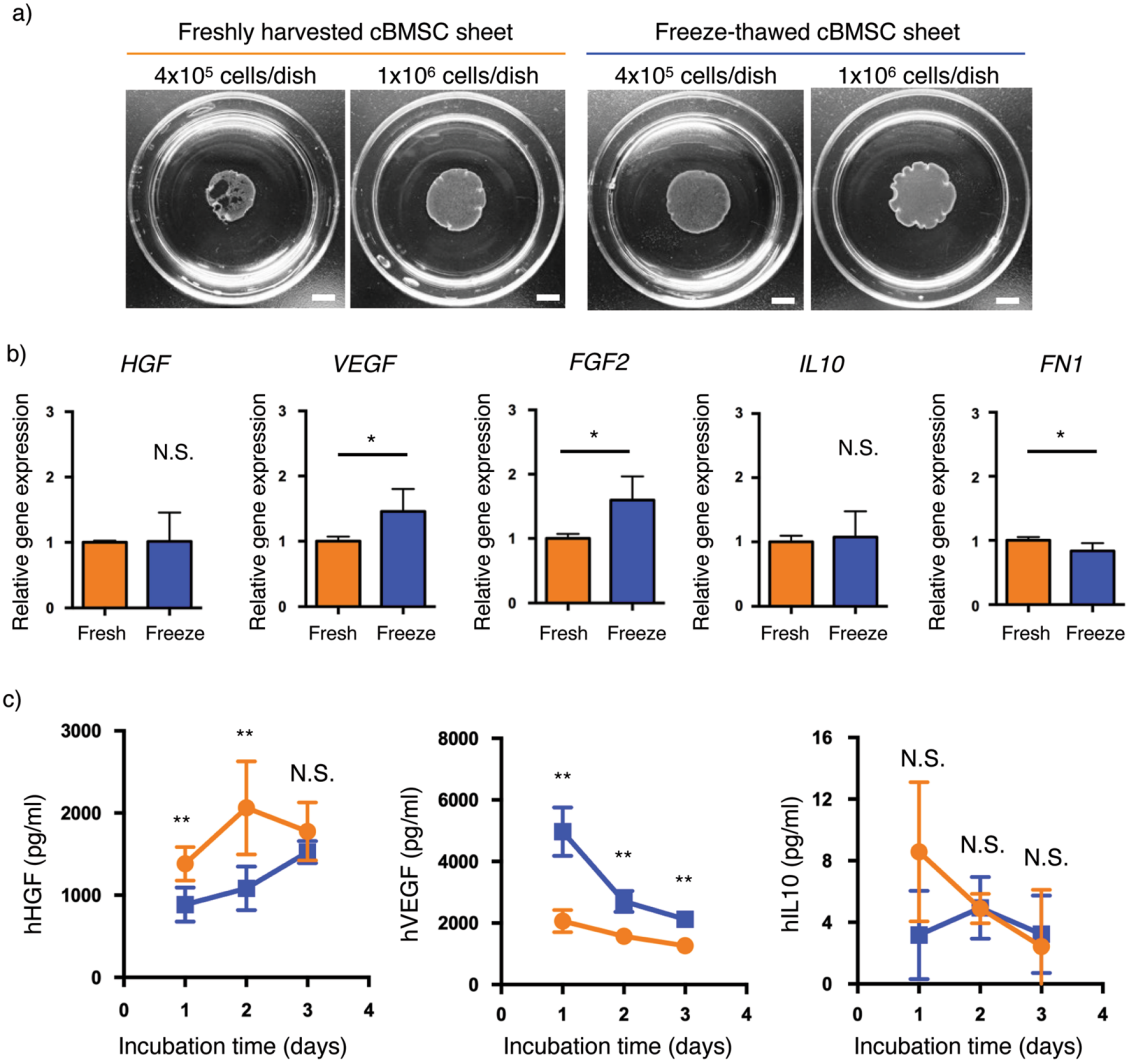
Cell sheet preparation using freshly harvested and freeze-thawed human clonal BMSC sheets as prepared in 24 h and comparison of cytokine production. (**a**) Macroscopic images of freshly harvested and freeze-thawed clonal BMSC sheets at the seeding densities of 4 × 10^5^ and 1 × 10^6^ cells/dish. Scale bars represent 500 mm. (**b**) Gene expression levels of *HGF*, *VEGF*, *FGF2*, *IL10*, and *FN1* in freshly harvested (fresh, orange) and freeze-thawed (freeze, blue) clonal BMSC sheets. Data shown are mean ± SD (n = 4). Statistical significance: Student’s *t*-test, not significant (N.S.) *P < 0.05. (**c**) Measurements of released cytokine amounts from freshly harvested (orange, n = 5) and freeze-thawed (blue, n = 4) clonal BMSC sheets. Data shown are mean ± SD (n = 4 or 5). Statistical significance: Student’s *t*-test, not significant (N.S.) **P < 0.01.

Current cell sheet preparation for allogeneic cell therapy requires approximately 2–3 weeks^19–21^ for cell expansion and maintenance when working from an established working cell bank without donor cell isolation. One approach to reduce the barriers to cell sheet applications is to simplify and shorten production steps to yield more cost-effective cell sheets fabricated for on-demand, acute or ready emergency use. To address the need for shorter, more cost-effective cell sheet fabrication methods, this study uses an extensive cell bank of long-term culture-adapted human cBMSCs to produce allogenic cell sheets within 24 h. Freshly harvested and freeze-thawed cBMSCs were used directly to prepare cBMSC sheets in 24 h using a high initial seeding cell density of 4 × 10^5^ and 1 × 10^6^ cells/dish (Fig. 5a).

We expected differences in rapid and functional cell sheet formation based on differences observed in adherent abilities of freshly harvested and freeze-thawed cBMSCs, specifically, increased cell sheet fabrication ability based on observed enhanced adhesion rates of freeze-thawed cBMSCs (see Figs. 3, 4). After 24-h incubation, cultured cells in each experimental group were successfully harvested from TRCDs as contiguous, contracted cell sheets via temperature-mediated detachment. As shown in Fig. 5a, freshly harvested cBMSCs produced a fragile cell sheet containing many defects/holes under the lower initial seeding density of 4 × 10^5^ cells/dish.

In contrast, freeze-thawed cBMSCs reliably produced intact cell sheets under the same 4 × 10^5^ (i.e., 0.4 M) cells/dish initial seeding density (Fig. 5a). At the higher initial seeding density of 1 × 10^6^ (1 M) cells/dish, both freshly harvested and freeze-thawed cBMSCs produced intact cell sheets (Fig. 5a). Resulting diameters of fresh and freeze-thawed harvested sheets (seeding: 1 M cells/35-mm dish) were 13.8 and 14.1 mm, respectively (n = 3/group, not statistically different). These results indicate that rapid (24 h) cell sheet formation is possible from freeze-thawed banked cBMSCs at multiple seeding densities, distinct from freshly harvested cBMSCs. Taken together, rapid adhesion of freeze-thawed cBMSCs may contribute to the enhanced ability to rapidly fabricate cell sheets, which is advantageous for reducing the time and costs of cell sheet production.

MSCs secrete multiple pro-regenerative cytokines (i.e., HGF, VEGF), making them an attractive alternative to single-molecule drugs in treating diseases such as renal fibrosis^45–48^. However, freeze-thawed MSCs have been reported to exhibit impaired cytokine production, specifically those related to immunomodulatory effects based on indoleamine 2,3-dioxygenase (IDO) expression^35,38,39^. Therefore, in this study, we sought to compare production of tissue-regenerative cytokines from freshly harvested and freeze-thawed cBMSC sheets at 1 × 10^6^ cells/dish seeding density. No differences in gene expression levels of HGF and IL-10 were observed between freshly harvested and freeze-thawed cBMSC sheets (Fig. 5b). Expression of VEGF and FGF2 was significantly higher, while FN was significantly lower in freeze-thawed cBMSC sheets, although these differences were slight (Fig. 5b). Overall, the cytokine production in freeze-thawed cBMSC sheets is comparable to freshly harvested cBMSC sheets, indicating no adverse effects of using freeze-thawed cBMSCs (Fig. 5b). We further evaluated the actual protein production from harvested cell sheets by replating detached cell sheets onto insert wells, incubating for 3 days, and collecting the media supernatant for ELISA assay. HGF concentration was significantly higher in freshly harvested cBMSC sheets at 1 and 2 days, but no differences were seen at 3 days (Fig. 5c). In contrast, VEGF concentration was significantly higher (around 2-fold higher) in freeze-thawed cBMSC sheets at each timepoint (Fig. 5c). No differences in IL-10 concentration were observed. These findings suggest that using freeze-thawed cBMSCs is a promising method to rapidly produce cell sheets with equivalent production of therapeutically relevant pro-regenerative cytokines to freshly harvested cBMSC sheets. This strategy could prove useful to suppress renal fibrosis, as shown in the rat IRI model previously^17,42^.

### Freeze-thawed cBMSC sheet transplantation in a rat ischemia–reperfusion injury (IRI) model to evaluate cell sheet therapeutic effects on acute renal fibrosis

**Figure 6:**
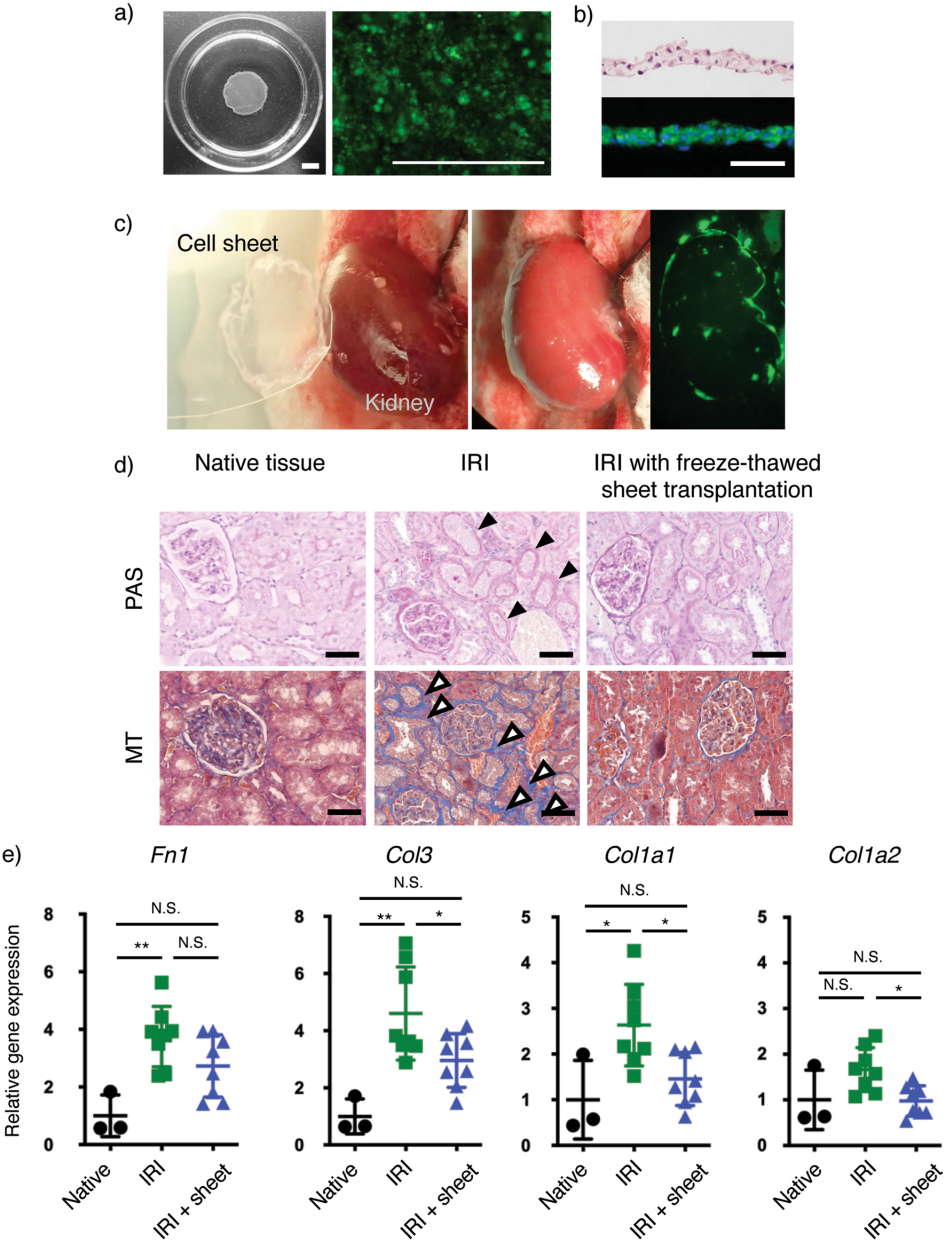
Therapeutic effects of rat freeze-thawed GFP-labeled clonal BMSC sheets in rat IRI model. (**a**) Macroscopic and fluorescent images of GFP-labeled rat freeze-thawed clonal BMSC sheets at 0-day harvest prior to cell sheet in vivo transplantation. Scale bars represent 500 μm. (**b**) Hematoxylin and eosin staining and GFP immunohistochemistry staining of rat freeze-thawed clonal BMSC sheets. Scale bars represent 50 μm. (**c**) Macroscopic and fluorescent images of cell sheet transplantation and attachment on rat kidney at 0-day cell sheet in vivo transplantation. (**d**) Periodic acid–Schiff (PAS) and Masson's trichrome staining. Black arrows indicate thick basement membrane. White arrows indicate fibrotic component deposition. Scale bars represent 50 μm. (**e**) Gene expression levels of fibrotic markers (*Fn1, Col3, Col1A1, Col1A2*) in kidneys collected from native (n = 3), IRI (n = 8), and IRI + freeze-thawed cBMSC sheet transplantation (n = 8) groups. Statistical significance: one-way ANOVA, Tukey’s multiple comparisons, not significant (N.S.) *P < 0.05, and **P < 0.01.

The efficacy of allogeneic freeze-thawed cBMSC sheets in preventing/treating renal fibrosis was determined previously by transplantation of freshly harvested rat BMSC sheets^17^ and cBMSC sheets^42^ using a published acute renal fibrosis rat IRI. In this study, to evaluate therapeutic efficacy of freeze-thawed cBMSC sheets, freeze-thawed GFP-labeled rat cBMSC sheets were fabricated by seeding cells onto TRCDs using the same established method as the human freeze-thawed cBMSC sheets. Sheet GFP fluorescent signal was observed in Fig. 6a,b just prior to kidney transplantation. The average diameter of freeze-thawed rat cBMSC sheets is analogous to sheets prepared from human BMSCs (approx. 1 cm, compare Figs. 5, 6), as shown in Fig. 6b. Freeze-thawed rat cBMSC sheets were transplanted directly to the renal capsule of the IRI kidney, as shown in Fig. 6b. Visualization of the cell sheet via GFP signal confirmed that transplanted cell sheets covered the entirety of the dorsal side and stably adhered to the kidney surface (Fig. 6b). On day 28 post-surgery, the kidneys were harvested for histological analysis. Disease progression of renal fibrosis was determined by ECM deposition and assessed using periodic acid-Schiff (PAS) and Masson’s trichrome (MT) staining (Fig. 6d). The rat IRI model without cell sheet transplantation (control, disease) group exhibited increased fibrotic area (indicated by the black arrowheads in Fig. 6d) compared to the cell sheet transplantation group. To quantify fibrotic components in respective kidneys, we investigated gene expression levels of fibronectin (*FN1*), collagen type 1 (*COL1A1, COL1A2*), and collagen type 3 (*COL3*). Importantly, expression levels of fibronectin (*FN1*) and collagen type 1 (*COL1A1, COL1A2*), common fibrotic tissue makers, were lower in the freeze–thaw cBMSC cell sheet transplantation group compared to IRI-only group (no cell sheet transplantation) (Fig. 6e). Additionally, collagen type 3 (*COL3*), a renal fibrosis marker, was significantly lower in the freeze–thaw cell sheet transplantation group compared to the IRI-only group (Fig. 6e). These findings suggest that allogeneic freeze-thawed cBMSC sheet transplantation suppresses renal fibrosis in the rat IRI model, demonstrating feasibility to address human fibrotic disease pathology, as indicated by reduced fibrotic areas.

## Discussion

Currently, there are many clinical trials investigating various uses of allogeneic MSCs to treat human disease and in regenerative medicine. However, unreliable critical quality attributes, lack of potency standards, insufficient product validation, and costly cell manufacturing costs remain critical barriers to clinical advancement of MSC-based cell therapies^49,50^. Progress with allogeneic MSC therapies requires improved, sustainable cell banking systems and efficient, cost-effective production methods that reliably and consistently yield a validated, potent cell therapy product. In this study, to reduce MSC sheet production time, we employed cryopreserved, freeze-thawed cBMSCs to shorten key cell sheet production steps from several weeks to 24 h (Fig. 5), facilitating urgent or emergency off-the-shelf use.

Freeze-thawed MSCs are the preferred cell preparation in clinical settings due to the convenience and accessibility, contributing to clinical and economic benefits^10,35,36,51,52^. However, clinical translation of freeze-thawed MSCs remains stalled by inconsistent efficacy, attributed to inappropriate optimization and criteria in using and validating properties of freeze-thawed cells^10,37,51,53^. Evaluation of GvHD clinical trial outcomes, defined as the loss of all symptoms or improvements against acute GvHD^54^, shows that patient benefits are doubled when using freshly isolated, non-cryopreserved MSCs cultured up to four passages compared to clinical studies using freeze-thawed MSCs^38^. Although general MSC phenotypic indicators, such as surface antigen expression and tri-lineage differentiation potency, are well-preserved in freeze-thawed MSCs^39,53^, low cell viability and impaired blood regulatory properties have been described following freeze–thaw cycles^38,52^. MSC immunomodulatory properties are also reported to be impaired after cryopreservation^35,38,39^ but can be rescued when exposed to IFN-γ for a 24-h culture^35^. Analogous IFN-γ priming of human cBMSCs to improve immunomodulatory factor productions in cell sheets similar to those reported here has recently been reported^55^.

In contrast to previous studies evaluating cryogenically preserved MSCs^10,35,36,51,52^, we have successfully engineered cBMSC sheets from freeze-thawed cells in 24 h with in vitro cytokine production comparable to freshly harvested cBMSC sheets (Fig. 5). This finding is different from previous contrasting studies reporting impaired functionality of freeze-thawed MSCs as single cells^10,35,36,51,52^. Furthermore, freeze-thawed cBMSC sheets demonstrate the therapeutic capacity to reduce renal fibrosis in a rat IRI kidney disease model (Fig. 6), similar to non-cryopreserved MSC sheets, as previously reported^17,42^. Thus, cell sheet technology may overcome the common disadvantages of freeze-thawed single cell MSC formulations as used in previous studies^10,35,36,51,52^. Our prior reports of cell sheet technology demonstrate that the MSC sheet three-dimensional (3D) structure enhances cytokine production compared to both single cells^20^ and 2D monolayer cultures before sheet detachment from cell cultureware^21^. Given supporting cell sheet data from these past studies, intrinsic three-dimensionality of freeze-thawed cBMSC sheets is reasonably inferred to contribute to production of multiple therapeutic cytokines (compare Figs. 5 and 6) and immunomodulatory factors^55^ that drive forward their therapeutic utility in kidney fibrosis models^17,42^.

Additionally, we found that freeze-thawed cBMSCs, compared to freshly harvested cBMSCs, exhibit greater initial cell adhesion and cell spreading ability after plating (t = 15 min) (Fig. 3a,b, supplemental Figure S1). Similarly, Pollock et al. showed that MSC cryopreservation in DMSO does not affect cell adhesion ability at 2 h post-plating^53^. However, MSCs incubated for 1-h in DMSO before freezing, exhibited a significantly reduced cell adhesion ability without affecting cell viability^53^. We believe this heightened cell spreading ability correlates to the observed improved rapid (24 h) cell sheet formation, enabling flexible cell sheet production from multiple different seeding densities ranging from low (0.4 × 10^6^ cells) to high (1 × 10^6^ cells) seeding densities per 35-mm TRCD dish (Fig. 5a). Cell adhesion molecule, ITGB1, expression was unchanged after freeze-thawing (Fig. 3c). Chinnadurai et al. showed that cell surface expression of adhesion molecules tetraspanin (CD63), integrin alpha V (CD51), MHC class I (HLA-ABC), integrin beta 1(CD29), integrin alpha 4 (CD49d), integrin alpha IIb (CD41), ICAM 1 (CD54), integrin beta 3 (CD61), and integrin alpha 5 (CD49e), do not differ between non-cryopreserved and freeze-thawed MSCs^36^. Taken together, this study shows that freeze-thawed cBMSCs possess greater initial cell adhesion ability compared to freshly harvested cBMSCs (Fig. 3a,b) while expression of cell adhesion molecule ITGB1 is comparable (Fig. 3c)^36,53^.

Increased cell spreading rate of freeze-thawed cBMSC, shown in Fig. 3, may be related to actin fiber formation associated with cell adhesion. Pollock et al.^36,53^ and Chinnadurai et al.^36,53^ demonstrate reduced formation of polymerized long-form actin stress fibers, F-actin, and elongated cells after freeze-thawing, also corroborated by our data (Fig. 4a–c). Instead of long-form actin fibers, freeze-thawed cBMSCs possess short-form actin fibers usually involved in early stages of cell adhesion (Fig. 4a–c), nascent adhesion, known as non-muscle myosin II-independent adhesion during cell spreading^56–59^. Notably, previous studies showed that mature, long actin fibers are implicated in focal adhesion contractile forces, reducing rates of cell spreading^60–62^. Therefore, it is reasonable to suggest that freeze-thawed cBMSCs exhibit enhanced cell spreading using short-form actin fibers, without mature actin fibers, compared to freshly harvested cBMSCs, advantageous for rapid cell sheet production. Additionally, due to lack of observed mature actin fibers in freeze-thawed cBMSCs, sizes of freeze-thawed cBMSC sheets are slightly larger, attributed to impaired actin fiber formation and thus reducing cell sheet contractile forces^21,63^ after cryopreservation without diminishing the sheet’s transplantability (Figs. 5a, 6c). Despite the observed lack of mature actin formation after freeze-thawing (Fig. 4)^36,53^, the actin reorganization pathway is reported to be upregulated in freeze-thawed cells^52^, a likely reason for why they adequately recover from cryo-induced stress and proliferate well after culture, as shown in Fig. 2b,c.

Variable MSC quality and biodistribution induced by freeze–thaw cycles reduces consistent and reliable cell systemic administration, complicating treatment of localized diseases and likely contributing to inconsistent and often insufficient therapeutic effects^10,30,35,36,38,51,52^. When MSCs are treated with cytochalasin D, a disruptor of actin fiber formation similar to that observed in freeze-thawed MSCs (Fig. 4), MSC in vivo biodistribution changes, instead engrafting largely in the lung and colon after systemic infusion and intraperitoneal injection, respectively^30,36,38^. Lack of actin fibers and unpredictable biodistribution of freeze-thawed MSCs may be responsible for insufficient clinical outcomes after systemic administration of freeze-thawed cBMSCs^10,38^. In contrast, retention of topically transplanted cell sheets is reported in many preclinical models of disease^22^. Cell sheets directly adhere and stably engraft to, and are retained on, targeted tissue sites spontaneously without suturing^17,18^. Cell sheet transplantation, a local MSC delivery method without systemic administration challenges, may overcome current issues associated with freeze-thawed cell suspensions to facilitate broader future clinical utility.

## Conclusions

This study reports successful MSC sheet production using long-term culture-adapted human and rat cBMSCs in both freshly harvested and freeze-thawed conditions. The high intrinsic cell spreading ability of freeze-thawed cBMSCs relates to accelerated cell sheet formation without compromising in vitro cytokine production and therapeutic ability to suppress renal fibrosis formation in vivo. Most investigators have focused on the disadvantages of freeze-thawed cells such as impaired immunomodulatory properties^35,38^ and biodistribution^30,36,38^. In this study, however, we show that cell sheet technology can overcome these limitations by fabricating cBMSC sheets generated directly from cryo-banked passaged stocks. Based on our recent reports showing immunomodulatory properties for cBMSC sheets^55^ and demonstrating therapeutic cBMSC sheet efficacy in the rodent IRI kidney fibrosis model^42^, we combined with the unique cBMSC sheet properties shown in this current study herein involving cryo-banked cell revival, expansion, rapid and reliable sheet formation and therapeutic cytokine production all fit a consistent picture for feasible future off-the-shelf, consistent clinical applications. This study proposes the strategic combination of clinical grade, long-term culture-adapted clonal BMSCs and cell sheet technology to improve therapeutic properties and promote further clinical translation of freeze-thawed MSCs. These findings represent an important first step for initiating use of rapidly fabricated cell sheets from freeze-thawed MSCs to treat renal fibrosis and possibly other organ pathologies in the future.

## Materials and methods

### Preparation of freshly harvested and freeze-thawed cBMSCs

Human clonal BMSC cell lines were provided by SCM Lifescience (Republic of Korea) for this study as reported^31^. Briefly, human bone marrow aspirate was mixed with growth media [Dulbecco's Modified Eagle's Medium (DMEM, Thermo Fisher Scientific, 11885076) supplemented with 20% Fetal Bovine Serum (FBS, Thermo Fisher Scientific, 16000044), 0.05% MycoZap Prophylactic (Lonza, VZA-2023), 1% penicillin streptomycin (Thermo Fisher Scientific, 15140163)] and cultured at 37 °C, 5% CO_2_ in a culture incubator for isolation of single-cell derived clonal BMSC cell line using their patented Subfractionation Culturing Method (SCM)^31^. Isolated human cBMSCs (clonal cell line: A106 D127, SCM Lifescience, Korea) were cultured in Dulbecco's Modified Eagle's Medium (DMEM, Thermo Fisher Scientific, 11885076) supplemented with 10% Fetal Bovine Serum (FBS, Thermo Fisher Scientific, 16000044), 0.05% MycoZap Prophylactic (Lonza, VZA-2023), 1% penicillin–streptomycin (Thermo Fisher Scientific, 15140163) at 37 °C, 5% CO_2_. Expanded cBMSCs were then collected using 0.05% trypsin EDTA (Thermo Fisher Scientific, 25200114), centrifuged at 210xg for 5 min, and resuspended in STEM-CELLBANKER cryogenic media (Amsbio, 11890) to establish working cell banks. cBMSCs in cryogenic media were then put in a freezing container (Corning, 432007) for 2 days at − 80 °C before being moved to a liquid nitrogen tank. Working cell banks were produced at both passage 8 (P8) and 10 (P10) (Fig. 1). Total cell number, cell viability, and doubling times of cultured cBMSCs were determined by trypan blue staining (MilliporeSigma, 72-57-1) and hemocytometers. MSC phenotypic validation of P10 cBMSCs was performed by cryopreserved cBMSCs at P8, culturing to P10 and testing for tri-lineage differentiation potential (osteogenesis, adipogenesis, and chondrogenesis), and surface antigen expression, CD73 + (BioLegend, 344014), CD90+ (BioLegend, 202507), CD105+ (BioLegend, 323208), CD44+ (BioLegend, 338808), CD34− (BioLegend, 343521), CD31− (BioLegend, 303110), CD45− (BioLegend, 304021) using flow cytometry (BD, Canto) (Supplemental Figure S1).

### Cell sheet preparation using freshly harvested and freeze-thawed cBMSC stocks

Freshly harvested cBMSC sheets were prepared by reviving cryopreserved cells from a P8 working cell bank in a 37 °C water bath more than 20 min, collecting the cells in a pre-warmed cell culture medium, and centrifuging at 210×g for 5 min. P8 cBMSCs were then seeded onto conventional cell culture flasks (CELLTREAT, 229351) and passaged twice prior to seeding harvested cells at P10 onto 35-mm temperature-responsive culture dishes (TRCDs, Thermo Fisher Scientific, 03150025) (Fig. 1). Freeze-thawed cBMSC sheets were prepared by reviving cryopreserved cells from the P10 working cell bank and seeding them directly onto a 35-mm TRCD without prior cultivation (Fig. 1). Both freshly harvested and freeze-thawed cBMSC sheets were prepared on TRCDs at seeding densities of 4 × 10^5^ and 1 × 10^6^ cells/dish and cultured for 24 h at 37 °C, 5% CO_2_ incubator in cell culture medium comprising DMEM (Thermo Fisher Scientific, 11885076) supplemented with 10% FBS (Thermo Fisher Scientific, 16000044), 0.05% MycoZap Prophylactic (Lonza, VZA-2023), 1% penicillin–streptomycin (Thermo Fisher Scientific, 15140163), and 50 µg/ml of l-ascorbic acid phosphate magnesium salt n-hydrate (Fujifilm Wako Pure Chemical, 013-19641).

### cBMSC in vitro adhesion assay

To investigate cell adhesion abilities under culture, single cell preparations of each experimental cBMSC group (i.e., freshly harvested and freeze-thawed stocks) were seeded onto 35-mm tissue culture treated dishes (CELLTREAT, 229635) at a seeding density of 5 × 10^4^ cells/dish. After 15–60 min, dishes were washed with PBS twice to remove any non-adherent cBMSCs and determine the number of adherent cells by counting the remaining adhered cells using phase contrast microscopy images (Zeiss, AXIOVert.A1, 5 random positions in each sample, n = 4). Additionally, adhesion rates of freshly harvested and freeze-thawed cBMSCs after seeding were observed using time-lapse imaging (Olympus, IV-83: n = 3) using a stage-top incubator (Tokai Hit, INU) and counting the number of adherent cells on 35-mm tissue culture treated dishes. Additionally, the gene expression of integrin β1 was examined at varying time points during cell adhesion and spreading. cBMSC were seeded onto 100-mm diameter dishes at 5000 cells/cm^2^ and incubated for 15, 30, and 60 min. After incubation, the dishes were washed with PBS twice, and total RNA was collected using RNeasy mini kits (Qiagen, 74104). cDNA synthesis was performed with 1 µg of total RNA using a High-Capacity cDNA Reverse Transcription Kit (Thermo Fisher Scientific, 4368814). Gene expression was examined by quantitative Real-Time PCR (qRT-PCR) using TaqMan^®^ Gene Expression Assays (Thermo Fisher Scientific: *ITGB1*; Hs01127536_m1), normalized to expression of the internal control gene, *B2M*, and compared between freshly harvested and freeze-thawed cBMSC sheets.

### Fluorescent microscopy analysis of cell actin fiber formation and focal adhesion molecule localization

Freshly harvested and freeze-thawed cBMSCs were each seeded onto FBS-coated chamber slides (CELLTREAT, 229164) at a seeding density of 2000 cells/cm^2^ to observe single cells, and incubated for 3, 5, and 24 h to follow their adherent cell morphology and focal adhesion formation using phalloidin and the focal adhesion marker, vinculin. At each time point, incubated cells were washed with PBS and fixed with 4% paraformaldehyde (PFA) for 10 min. Samples were then incubated with 5% goat serum (Jackson Immunoresearch, 005-000-121) and 0.1% Triton X (Sigma-Aldrich, T8787) in PBS for 1 h at room temperature, followed by incubation with primary antibody, anti-human vinculin (MilliporeSigma, V9131), for 2 h at room temperature. After washing with PBS for 30 min, samples were incubated with secondary antibody, anti-mouse IgG Alexa Fluor 568 (Thermo Fisher Scientific, A-11004), and Alexa Fluor 488 phalloidin (Thermo Fisher Scientific, A12379) for 1 h at room temperature. Stained samples were mounted with ProLong™ Gold Antifade Mountant with DAPI (Thermo Fisher Scientific, P36935). Images were taken by a fluorescent microscope (Zeiss, AXIOVert.A1: 5 random positions in each sample, n = 4), and fluorescent signal areas were quantified by Image J (National Institutes of Health). Feret’s diameters, a measure of the longest and shortest diameter of adherent cells, were measured by image J to quantify cell shapes^43,44^. Error bars indicate standard deviations.

### Cytokine production analysis

Total RNA from cBMSC sheets was collected, and cDNA synthesis was performed as described above. Gene expressions were examined by qRT-PCR using TaqMan^®^ Gene Expression Assays (Thermo Fisher Scientific: *B2M*; Hs00187842_m1, *HGF*; Hs00379140_m1, *VEGFA*; Hs99999070_m1, *FGF2*; Hs00266645_m1, *IL10*; Hs00961622_m1, *FN1*; Hs01549976_m1), normalized to the expression level of the internal control gene, *B2M*, and compared between freshly harvested and freeze-thawed cBMSC sheets. In addition, specific protein production was measured by evaluating protein concentrations in the media using ELISA (R&D Systems; Human HGF Quantikine ELISA Kit; DHG00B, Human VEGF Quantikine ELISA Kit; DVE00, Human IL-10 Quantikine ELISA Kit; D1000B). To investigate cytokine release from fabricated cell sheets detached freshly harvested and freeze-thawed cBMSC sheets were replated onto 6-well inserts and incubated for 3-days in cell culture media with supernatant collection each day.

### Freeze-thawed rat BMSC sheet transplantation in rodent kidney IRI model

Rat cBMSCs derived from SDTg (CAG-EGFP) rats were provided by SCM Lifescience (Republic of Korea) and confirmed for rat MSC phenotype by tri-lineage differentiation and surface maker expression of CD90+, CD29+, MHC class II−, CD11−, and CD45− at P5. Rat freeze-thawed cBMSC sheets derived from SDTg (CAG-EGFP) rats were prepared following protocols identical to the human freeze-thawed cBMSC sheets described above. The rat kidney fibrosis model study was conducted under approval of the Animal Care & Use Committee, IACUC, University of Utah (assigned ID: 19-03011). All experiments were conducted in accordance with relevant guidelines and regulations. Study procedures were reported previously for the rat BMSC sheet transplantation model^17^, except in this study, the renal capsule remained intact, and no right kidney nephrectomy occurred^42^. Briefly, Lewis rats (6-week-old, males, Charles River Laboratories) were acclimatized in facilities for one week and randomly divided into three groups: (1) native tissue (n = 3), (2) IRI procedure without freeze-thawed cBMSC sheet transplantation (n = 8) and (3) IRI with freeze-thawed cBMSC sheet transplantation (n = 8), with no animal exclusions. Under isoflurane anesthesia, the IRI model was performed by clamping the left renal pedicle for 60 min. Allogeneic rat cBMSC GFP-cell sheets were transplanted onto the intact left renal capsule; transplanted cell sheets covered the kidney dorsal side and stably adhered to the entire kidney surface without suturing. At 4-weeks post-surgery, all kidneys were collected for histological analysis using periodic acid–Schiff (PAS) and Masson's trichrome (MT) staining to evaluate excess extracellular matrix deposition indicative of acute renal fibrosis. Additionally, the renal parenchymas were collected from each rat for gene expression analysis to evaluate fibrotic marker expression. Tissue homogenization by forceps mincing and syringing allowed total RNA extraction using RNeasy Fibrous Tissue Mini Kits (Qiagen; 74704). cDNA synthesis was performed as described above. Gene expression was examined by qRT-PCR using TaqMan^®^ Gene Expression Assays and normalized to expression levels of the internal control gene, *B2m*, and compared between freshly harvested and freeze-thawed cBMSC sheets. One surgical and non-surgical, blinded, operators performed analysis separately. All procedures were performed in accordance with ARRIVE guidelines.

### Statistical analysis

All statistical analysis for in vitro experiments was conducted with data sets of n = 4 (Figs. 2, 3, 4, 5b) and n = 4 or 5 (Fig. 5c) using unpaired, two-tailed, Student’s *t-*test. Different statistical analysis for in vivo experiments was conducted with data sets of native (n = 3), IRI (n = 8), IRI+ freeze-thawed cBMSC sheet transplantation (n = 8) groups (Fig. 6e). Statistical analysis was conducted by one-way ANOVA, with Tukey’s multiple comparisons. Statistical significance was defined as **P < 0.01, *P < 0.05 and not significant (N.S.) P ≥ 0.05 using GraphPad Prism (http://www.graphpad.com).

## Supplementary Information

Supplementary Information 1.

Supplementary Information 2.

Supplementary Video 1.

Supplementary Video 2.

## Data Availability

Data sets generated in this study are available from the corresponding author upon reasonable request.

## References

[1] Semedo P (2009). Mesenchymal stem cells attenuate renal fibrosis through immune modulation and remodeling properties in a rat remnant kidney model. Stem Cells.

[2] Xu SG, Liu C, Ji HL (2019). Concise review: Therapeutic potential of the mesenchymal stem cell derived secretome and extracellular vesicles for radiation-induced lung injury: Progress and hypotheses. Stem Cells Transl. Med..

[3] Németh K (2009). Bone marrow stromal cells attenuate sepsis via prostaglandin E 2-dependent reprogramming of host macrophages to increase their interleukin-10 production. Nat. Med..

[4] Mizuno S (1998). Hepatocyte growth factor prevents renal fibrosis and dysfunction in a mouse model of chronic renal disease. J. Clin. Investig..

[5] Kawaida K, Matsumoto K, Shimazu H, Nakamura T (1994). Hepatocyte growth factor prevents acute renal failure and accelerates renal regeneration in mice. Proc. Natl. Acad. Sci. USA.

[6] Yu MA (2009). HGF and BMP-7 ameliorate high glucose-induced epithelial-to-mesenchymal transition of peritoneal mesothelium. J. Am. Soc. Nephrol..

[7] Yuan L (2011). VEGF-modified human embryonic mesenchymal stem cell implantation enhances protection against cisplatin-induced acute kidney injury. Am. J. Physiol. Renal Physiol..

[8] Togel F (2007). Vasculotropic, paracrine actions of infused mesenchymal stem cells are important to the recovery from acute kidney injury. Am. J. Physiol. Renal Physiol..

[9] Togel F, Zhang P, Hu Z, Westenfelder C (2009). VEGF is a mediator of the renoprotective effects of multipotent marrow stromal cells in acute kidney injury. J. Cell. Mol. Med..

[10] Galipeau J, Sensebe L (2018). Mesenchymal stromal cells: Clinical challenges and therapeutic opportunities. Cell Stem Cell.

[11] Makhija R, Kingsnorth AN (2002). Cytokine storm in acute pancreatitis. J. Hepatobil. Pancreat. Surg..

[12] Devine SM, Cobbs C, Jennings M, Bartholomew A, Hoffman R (2003). Mesenchymal stem cells distribute to a wide range of tissues following systemic infusion into nonhuman primates. Blood.

[13] Zhu XY, Lerman A, Lerman LO (2013). Concise review: Mesenchymal stem cell treatment for ischemic kidney disease. Stem Cells.

[14] Kushida A (1999). Decrease in culture temperature releases monolayer endothelial cell sheets together with deposited fibronectin matrix from temperature-responsive culture surfaces. J. Biomed. Mater. Res..

[15] Okano T, Yamada N, Okuhara M, Sakai H, Sakurai Y (1995). Mechanism of cell detachment from temperature-modulated, hydrophilic-hydrophobic polymer surfaces. Biomaterials.

[16] Yamato M, Okano T (2004). Cell sheet engineering. Mater. Today.

[17] Imafuku A (2019). Rat mesenchymal stromal cell sheets suppress renal fibrosis via microvascular protection. Stem Cells Transl. Med..

[18] Sekine H (2011). Cardiac cell sheet transplantation improves damaged heart function via superior cell survival in comparison with dissociated cell injection. Tissue Eng. Part A.

[19] Kim K, Bou-Ghannam S, Thorp H, Grainger DW, Okano T (2019). Human mesenchymal stem cell sheets in xeno-free media for possible allogenic applications. Sci. Rep..

[20] Nakao M (2019). Phenotypic traits of mesenchymal stem cell sheets fabricated by temperature-responsive cell culture plate: Structural characteristics of MSC sheets. Stem Cell. Res. Ther..

[21] Bou-Ghannam S, Kim K, Grainger DW, Okano T (2021). 3D cell sheet structure augments mesenchymal stem cell cytokine production. Sci. Rep..

[22] Kondo M, Kameishi S, Grainger DW, Okano T (2020). Novel therapies using cell sheets engineered from allogeneic mesenchymal stem/stromal cells. Emerg. Top. Life Sci..

[23] Miyahara Y (2006). Monolayered mesenchymal stem cells repair scarred myocardium after myocardial infarction. Nat. Med..

[24] Iwata T (2018). Periodontal regeneration with autologous periodontal ligament-derived cell sheets—a safety and efficacy study in ten patients. Regener. Ther..

[25] Iwata T (2009). Periodontal regeneration with multi-layered periodontal ligament-derived cell sheets in a canine model. Biomaterials.

[26] Kaibuchi N, Iwata T, Yamato M, Okano T, Ando T (2016). Multipotent mesenchymal stromal cell sheet therapy for bisphosphonate-related osteonecrosis of the jaw in a rat model. Acta Biomater..

[27] Kato Y (2015). Allogeneic transplantation of an adipose-derived stem cell sheet combined with artificial skin accelerates wound healing in a rat wound model of type 2 diabetes and obesity. Diabetes.

[28] Ryu B (2019). Allogeneic adipose-derived mesenchymal stem cell sheet that produces neurological improvement with angiogenesis and neurogenesis in a rat stroke model. J. Neurosurg..

[29] Levy O (2020). Shattering barriers toward clinically meaningful MSC therapies. Sci. Adv..

[30] Giri J, Galipeau J (2020). Mesenchymal stromal cell therapeutic potency is dependent upon viability, route of delivery, and immune match. Blood Adv..

[31] Song SU (2008). Variations of clonal marrow stem cell lines established from human bone marrow in surface epitopes, differentiation potential, gene expression, and cytokine secretion. Stem Cells Dev..

[32] Kim M (2018). Transplantation of human bone marrow-derived clonal mesenchymal stem cells reduces fibrotic scar formation in a rat spinal cord injury model. J. Tissue Eng. Regen. Med..

[33] Ma A (2013). Reconstruction of cartilage with clonal mesenchymal stem cell-acellular dermal matrix in cartilage defect model in nonhuman primates. Int. Immunopharmacol..

[34] Yi T (2015). Manufacture of clinical-grade human clonal mesenchymal stem cell products from single colony forming unit-derived colonies based on the subfractionation culturing method. Tissue Eng. Part C Methods.

[35] François M (2012). Cryopreserved mesenchymal stromal cells display impaired immunosuppressive properties as a result of heat-shock response and impaired interferon-γ licensing. Cytotherapy.

[36] Chinnadurai R (2014). Actin cytoskeletal disruption following cryopreservation alters the biodistribution of human mesenchymal stromal cells in vivo. Stem Cell Rep..

[37] Bahsoun S, Coopman K, Akam EC (2019). The impact of cryopreservation on bone marrow-derived mesenchymal stem cells: A systematic review. J. Transl. Med..

[38] Moll G (2014). Do cryopreserved mesenchymal stromal cells display impaired immunomodulatory and therapeutic properties?. Stem Cells.

[39] Oja S (2019). The utilization of freezing steps in mesenchymal stromal cell (MSC) manufacturing: Potential impact on quality and cell functionality attributes. Front. Immunol..

[40] Jung KH (2011). Human bone marrow-derived clonal mesenchymal stem cells inhibit inflammation and reduce acute pancreatitis in rats. Gastroenterology.

[41] Yi HG (2016). Allogeneic clonal mesenchymal stem cell therapy for refractory graft-versus-host disease to standard treatment: A phase I study. Korean J. Physiol. Pharmacol..

[42] Oka M (2023). Clinically relevant mesenchymal stem/stromal cell sheet transplantation method for kidney disease. Tissue Eng. Part C Methods.

[43] Konstankiewicz K, Pawlak K, Zdunek A (2001). Quantitative method for determining cell structural parameters of plant tissues. Int. Agrophys..

[44] Baskaran JP (2020). Cell shape, and not 2D migration, predicts extracellular matrix-driven 3D cell invasion in breast cancer. APL Bioeng..

[45] Nangaku M (2006). Chronic hypoxia and tubulointerstitial injury: A final common pathway to end-stage renal failure. J. Am. Soc. Nephrol..

[46] Oka M, Sekiya S, Sakiyama R, Shimizu T, Nitta K (2019). Hepatocyte growth factor-secreting mesothelial cell sheets suppress progressive fibrosis in a rat model of CKD. J. Am. Soc. Nephrol..

[47] Mizuno S, Matsumoto K, Nakamura T (2001). Hepatocyte growth factor suppresses interstitial fibrosis in a mouse model of obstructive nephropathy. Kidney Int..

[48] Tögel F (2009). Autologous and allogeneic marrow stromal cells are safe and effective for the treatment of acute kidney injury. Stem Cells Dev..

[49] Olsen TR, Ng KS, Lock LT, Ahsan T, Rowley JA (2018). Peak MSC-are we there yet?. Front. Med. (Lausanne).

[50] Pereira Chilima TD, Moncaubeig F, Farid SS (2018). Impact of allogeneic stem cell manufacturing decisions on cost of goods, process robustness and reimbursement. Biochem. Eng. J..

[51] Galipeau J (2013). The mesenchymal stromal cells dilemma—does a negative phase III trial of random donor mesenchymal stromal cells in steroid-resistant graft-versus-host disease represent a death knell or a bump in the road?. Cytotherapy.

[52] Hoogduijn MJ (2016). Effects of freeze–thawing and intravenous infusion on mesenchymal stromal cell gene expression. Stem Cells Dev..

[53] Pollock K (2017). Improved post-thaw function and epigenetic changes in mesenchymal stromal cells cryopreserved using multicomponent osmolyte solutions. Stem Cells Dev..

[54] Le Blanc K (2008). Mesenchymal stem cells for treatment of steroid-resistant, severe, acute graft-versus-host disease: A phase II study. Lancet.

[55] Dunn CM (2022). Interferon-gamma primed human clonal mesenchymal stromal cell sheets exhibit enhanced immunosuppressive function. Cells.

[56] Choi CK (2008). Actin and α-actinin orchestrate the assembly and maturation of nascent adhesions in a myosin II motor-independent manner. Nat. Cell Biol..

[57] Vicente-Manzanares M, Ma X, Adelstein RS, Horwitz AR (2009). Non-muscle myosin II takes centre stage in cell adhesion and migration. Nat. Rev. Mol. Cell Biol..

[58] Alexandrova AY (2008). Comparative dynamics of retrograde actin flow and focal adhesions: Formation of nascent adhesions triggers transition from fast to slow flow. PLoS One.

[59] Brieher W (2013). Mechanisms of actin disassembly. Mol. Biol. Cell.

[60] Takizawa N, Ikebe R, Ikebe M, Luna EJ (2007). Supervillin slows cell spreading by facilitating myosin II activation at the cell periphery. J. Cell Sci..

[61] Cai Y (2006). Nonmuscle myosin IIA-dependent force inhibits cell spreading and drives F-actin flow. Biophys. J..

[62] Lo C-M (2004). Nonmuscle myosin IIb is involved in the guidance of fibroblast migration. Mol. Biol. Cell.

[63] Thorp H, Kim K, Kondo M, Grainger DW, Okano T (2020). Fabrication of hyaline-like cartilage constructs using mesenchymal stem cell sheets. Sci. Rep..

