# Supplementary material for: Rapid and effective preparation of clonal bone marrow-derived mesenchymal stem/stromal cell sheets to reduce renal fibrosis
Source: Sci Rep. 2023 Mar 17;13:4421. doi: 10.1038/s41598-023-31437-7 (PMC10023793; doi:10.1038/s41598-023-31437-7)
Supplement: Supplementary file 1 — Supplementary Information 1. [file 41598_2023_31437_MOESM1_ESM.pdf]

Rapid and effective preparation of clonal bone marrow-derived mesenchymal stem/stromal cell sheets to reduce renal fibrosis

Sumako Kameishi, Celia M. Dunn, Masatoshi Oka, Kyungsook Kim, Yun-Kyoung Cho, Sun U Song, David W. Grainger, and Teruo Okano

Supplementary video legends

Supplementary Video 1: Cell adhesion of freshly harvested cBMSC

The cell behavior of freshly harvested cBMSCs during initial cell adhesion was investigated using time-laps imaging (n = 3).

Supplementary Video 2: Cell adhesion of freeze-thawed cBMSC

The cell behavior of freeze-thawed cBMSCs during initial cell adhesion was investigated using time-laps imaging (n = 3).
