# Supplementary material for: Rapid and effective preparation of clonal bone marrow-derived mesenchymal stem/stromal cell sheets to reduce renal fibrosis
Source: Sci Rep. 2023 Mar 17;13:4421. doi: 10.1038/s41598-023-31437-7 (PMC10023793; doi:10.1038/s41598-023-31437-7)
Supplement: Supplementary file 2 — Supplementary Information 2. [file 41598_2023_31437_MOESM2_ESM.pdf]

Rapid and effective preparation of clonal bone marrow-derived mesenchymal stem/stromal cell sheets to reduce renal fibrosis

Sumako Kameishi[1,2]*, Celia M. Dunn[1,3], Masatoshi Oka[1,2], Kyungsook Kim[1,2], Yun-Kyoung Cho[4], Sun U Song[4], David W. Grainger[1,2,3], and Teruo Okano[1,2,5]*

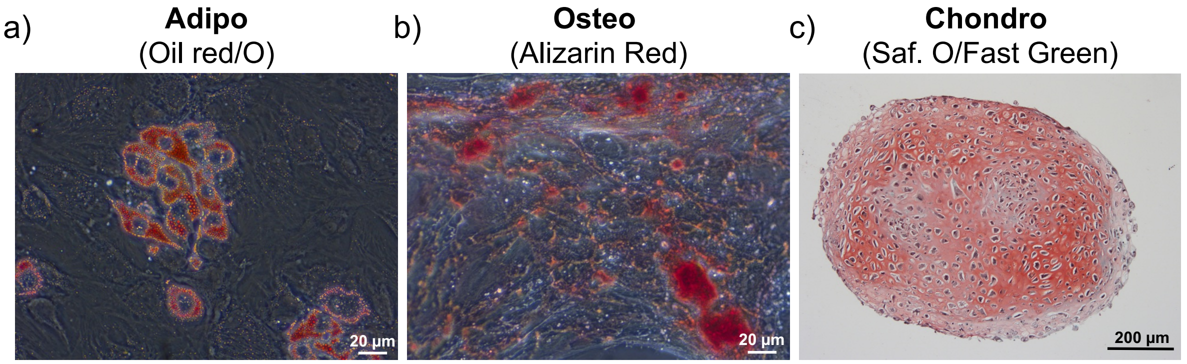

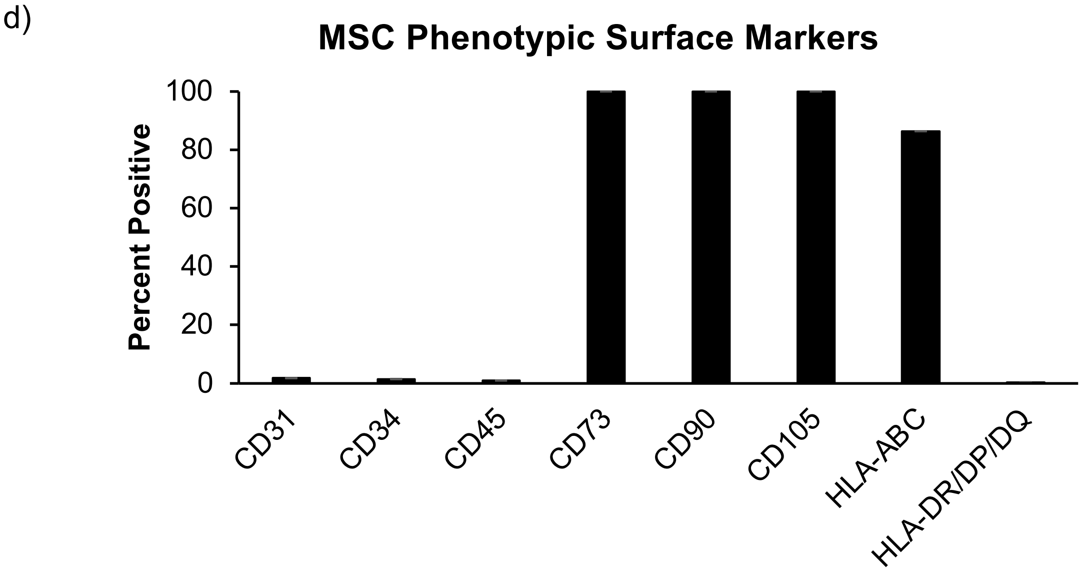

Supplemental Figure S1. Tri-lineage differentiation potency and MSC surface marker expressions at passage 10

(a-c) microscopic images of MSC trilineage differentiation, (a) adipogenesis (Oil red/O staining), (b) osteogenesis (Alizarin Red staining), and (c) chondrogenesis (Safranin O, Fast Green staining). (d) surface antigen expression showing positive expression of MSC markers CD73, CD90, CD105, and negative expression of hematopoietic markers CD31, CD34, CD45.

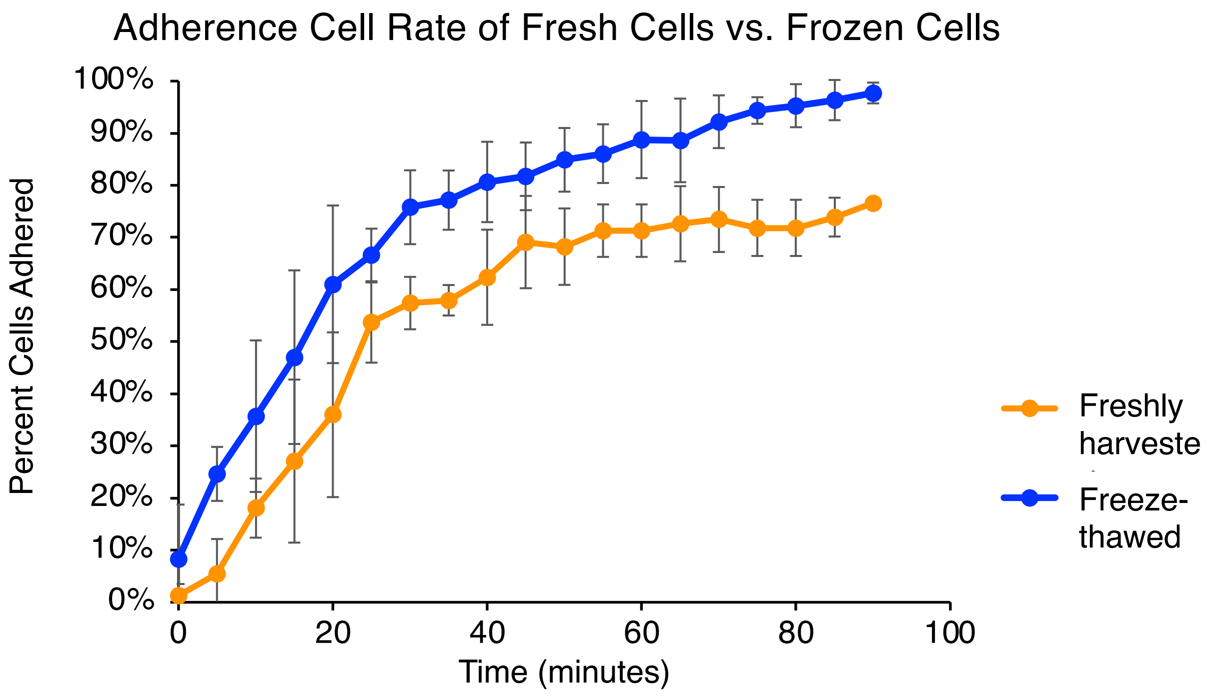

Supplemental Figure S2. Cell adhesion behavior of fresh and frozen BMSCs

Adherent cell rate of freshly harvested and freeze-thawed are investigated using time-laps imaging.
